# The Partial Support of the Left Ventricular Assist Device Shifts the Systemic Cardiac Output Curve Upward in Proportion to the Effective Left Ventricular Ejection Fraction in Pressure-Volume Loop

**DOI:** 10.3389/fcvm.2020.00163

**Published:** 2020-09-15

**Authors:** Takamori Kakino, Keita Saku, Takuya Nishikawa, Kenji Sunagawa

**Affiliations:** ^1^Department of Cardiology, St.Mary's Hospital, Kurume, Japan; ^2^Department of Cardiovascular Dynamics, National Cerebral and Cardiovascular Center Research Institute, Osaka, Japan; ^3^Department of Cardiovascular Medicine, Graduate School of Medical Sciences, Kyushu University, Fukuoka, Japan; ^4^Circulatory System Research Foundation, Fukuoka, Japan

**Keywords:** left ventricular assist device (LVAD), hemodymamics, circulatory equilibrium, prediction, pressure volume loop, impella

## Abstract

Left ventricular assist device (LVAD) has been saving many lives in patients with severe left ventricular (LV) failure. Recently, a minimally invasive transvascular LVAD such as Impella enables us to support unstable hemodynamics in severely ill patients. Although LVAD support increases total LV cardiac output (CO_TLV_) at the expense of decreases in the native LV cardiac output (CO_NLV_), the underlying mechanism determining CO_TLV_ remains unestablished. This study aims to clarify the mechanism and develop a framework to predict CO_TLV_ under known LVAD flow (CO_LVAD_). We previously developed a generalized framework of circulatory equilibrium that consists of the integrated CO curve and the VR surface as common functions of right atrial pressure (P_RA_) and left atrial pressure (P_LA_). The intersection between the integrated CO curve and the VR surface defines circulatory equilibrium. Incorporating LVAD into this framework indicated that LVAD increases afterload, which in turn decreases CO_NLV_. The total LV cardiac output (CO_TLV_) under LVAD support becomes CO_TLV_ = CO_NLV_+EF_e_ · CO_LVAD_, where EF_e_ is effective ejection fraction, i.e., E_es_/(E_es_+E_a_). E_es_ and E_a_ represent LV end-systolic elastance (E_es_) and effective arterial elastance (E_a_), respectively. In other words, LVAD shifts the total LV cardiac output curve upward by EF_e_ · CO_LVAD_. In contrast, LVAD does not change the VR surface or the right ventricular CO curve. In six anesthetized dogs, we created LV failure by the coronary ligation of the left anterior descending artery and inserted LVAD by withdrawing blood from LV and pumping out to the femoral artery. We determined the parameters of the CO curve with a volume-change technique. We then changed the CO_LVAD_ stepwise from 0 to 70–100 ml/kg/min and predicted hemodynamics by using the proposed circulatory equilibrium. Predicted CO_TLV_, P_RA_, and P_LA_ for each step correlated well with those measured (SEE; 2.8 ml/kg/min 0.17 mmHg, and 0.65 mmHg, respectively, r^2^; 0.993, 0.993, and 0.965, respectively). The proposed framework quantitatively predicted the upward-shift of the total CO curve resulting from the synergistic effect of LV systolic function and LVAD support. The proposed framework can contribute to the safe management of patients with LVAD.

## Introduction

Heart failure is one of the most challenging cardiac pathophysiologies, and the survival rate remains unacceptably poor despite the guideline-recommended optimal medical therapy (1). Although heart transplantation strikingly improves the quality of life and prolongs survival in patients with end-stage heart failure, the number of donor's hearts is disproportionally small (2). Therefore, heart transplantation cannot serve as a standard therapeutic modality for every patient with end-stage heart failure. Left ventricular assist device (LVAD) has been saving many lives as a bridge to recovery, transplantation, and decision (3–5). Klotz et al. reported that, even in end-stage heart failure, LVAD could reverse ventricular remodeling. They argued that mechanical LV unloading improves neurohormonal/cytokine milieu and reverses LV remodeling (6).

The latest advance in medical technology has allowed us to develop minimally invasive transvascular LVAD such as Impella® (Abiomed Inc. Danvers, MA, USA). The fact that LVAD promotes recovery of myocardial function makes temporary LVAD implantation as a practical therapeutic option in the treatment of heart failure (7). In myocardial infarction, transvascular LVAD reduces infarct size and promotes LV recovery (8, 9). In fulminant myocarditis, transvascular LVAD helps to suppress inflammation and facilitate recovery (10). Considering those devices development, the appropriate LVAD use improves the outcome of heart failure patients in several stages.

Hemodynamic responses to the “off-pump” trial were critical in weaning LVAD and predicting long-term cardiac stability after weaning (11). Therefore, the prediction of the hemodynamic impact of LVAD support and explantation is a prerequisite in the safety management of hemodynamically compromised patients. We previously reported the impact of total LVAD support, i.e., no LV ejection through the aortic valve, on hemodynamics by using the framework of circulatory equilibrium in an animal model of acute heart failure (12). We could successfully predict total LVAD induced changes in hemodynamics. However, the recovery of LV function increased LV contractility and makes LVAD support partial, i.e., significant LV ejection through the aortic valve. How to predict the hemodynamics of partial LVAD support remains unknown.

This study aims to develop a framework to predict the impact of partial LVAD support on hemodynamics. To answer this complex question, we first analyzed the quantitative effect of partial LVAD support on the LV pressure-volume relationship by using the concept of the left ventricular-arterial coupling (13). We then incorporated the ventricular-arterial coupling into the framework of circulatory equilibrium and predicted hemodynamics. Finally, we compared the predicted hemodynamic variables with those measured in an animal model of heart failure.

## Theoretical Consideration

### Circulatory Equilibrium

In the 1950s, Guyton proposed a disruptive concept, the framework of circulatory equilibrium, because the CO curve alone could not determine cardiac output in the closed-loop circulation (14). They opened the circulatory loop and represented the venous returning (VR) curve and the CO curve as a function of right atrial pressure (P_RA_). They defined the circulatory equilibrium by the intersection between the CO curve and the VR curve. Although this framework explains numerous pathophysiological conditions such as volume overload, heart failure, and exercise, they failed to express unilateral heart failure and resultant volume redistribution between the systemic circulation and the pulmonary circulation. This inability of the Guyton's framework makes its application seriously limited.

To overcome the limitations of Guyton's circulatory equilibrium, we developed a generalized framework of circulatory equilibrium that consists of the integrated CO curve and the VR surface as common functions of P_RA_ and left atrial pressure (P_LA_) (15). In this framework, the intersecting curve between the two surfaces, systemic and pulmonary CO surfaces defines the integrated CO curve. The integrated CO curve can separately represent the left and right ventricular pumping function. The VR surface has two slopes along P_LA_ and P_RA_ axes, which represent vascular properties. We experimentally validated the flatness of the VR surface and demonstrated that the changes in stressed blood volume shift the VR surface in parallel along the VR axis. The VR surface allows us to express the redistribution of stressed blood volume between the systemic circulation and the pulmonary circulation resulting from unilateral heart failure. The intersection between the integrated CO curve and the VR surface represents the generalized circulatory equilibrium and defines the operating points of CO, P_RA_, and P_LA_.

### The Impact of LVAD on the LV Cardiac Output (CO_LV_) Curve

In the systemic circulation, the effect of downstream pressure, P_RA_, on CO_LV_ is negligible because P_RA_ is much lower than systemic arterial pressure. Therefore, we described the CO_LV_ as the curve, not the surface.

As explained in Supplementary Material, a logarithmic function of P_LA_ approximates the native CO curve without LVAD (CO_NLV_) as

(1) $$\begin{array}{l} CO_{NLV}=S_{L}\left\{ln\left(P_{LA}\right)+H_{L}\right\} \end{array}$$

where S_L_ and H_L_ represent parameters of the left heart.

**Figure 1:**
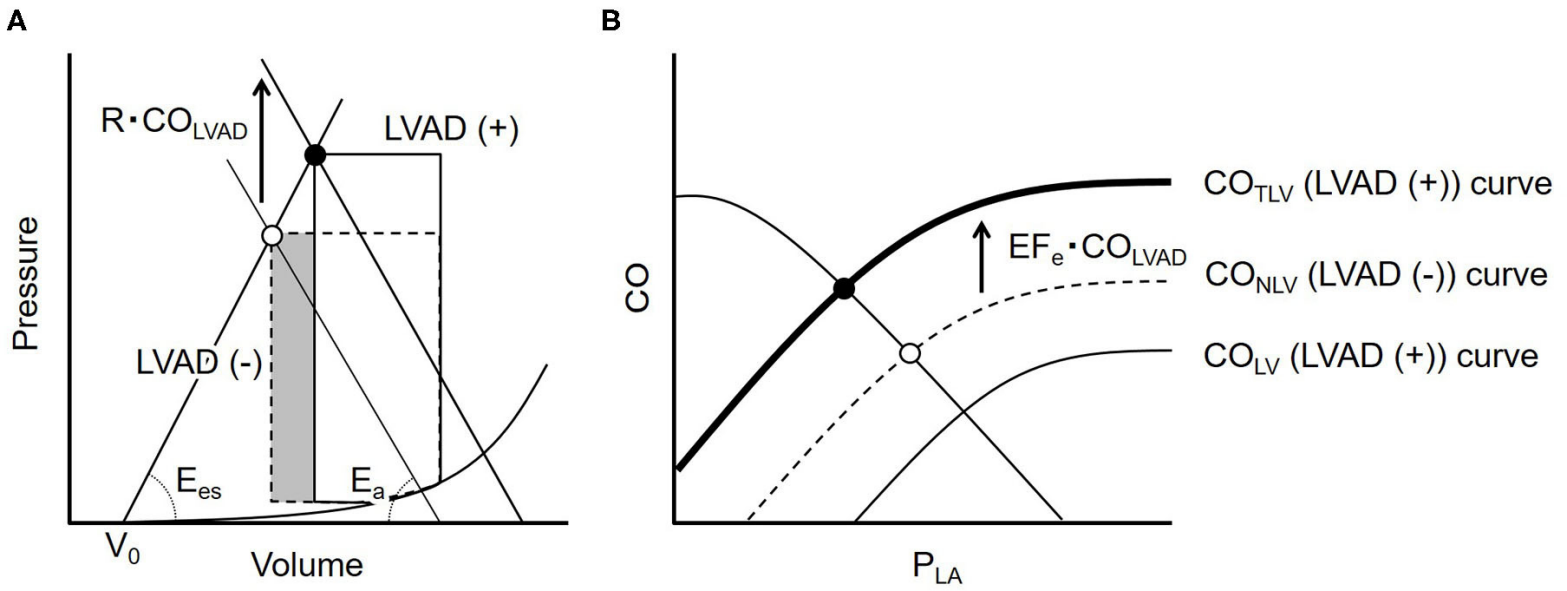
**(A)** The ventricular-arterial coupling in the pressure-volume trajectory. The dashed line represents the PV loop at baseline. LVAD flow (CO_LVAD_) shifts the effective arterial elastance (E_a_) line upward by R · CO_LVAD_ and moves the end-systolic point upward from the open circle to the solid one (solid loop). The volume of the shaded part indicates the LVAD induced decrease in stroke volume. **(B)** The dashed curve illustrates the native LV cardiac output (CO_NLV_) curve without LVAD. CO_LVAD_ decreases the CO_NLV_ curve downward (thin solid line = CO_LV_ curve), but increases the total CO (CO_TLV_) curve, the sum of the CO_LV_ and CO_LVAD_, indicating that LVAD shifts the CO_NLV_ curve upward by EF_e_ · CO_LVAD_ (bold solid line). LVAD, left ventricular assist device; CO_LVAD_, LVAD flow; E_es_, end-systolic elastance; E_a_, effective arterial elastance; R, systemic vascular resistance; V_0_, volume axis intercept of LV end-systolic pressure-volume relationship; CO_NLV_, native LV cardiac output through the aortic valve; CO_LV_, LV cardiac output through the aortic valve under LVAD; CO_TLV_, total LV cardiac output; P_LA_, left atrial pressure; P_RA_, right atrial pressure; EF_e_, effective ejection fraction.

Figure 1 illustrates the impact of LVAD on stroke volume (SV) on the LV pressure-volume relationship. As shown in the dashed loop in Figure 1A, the intersection between the end-systolic pressure-volume relationship line and the effective arterial elastance (E_a_) line determines SV for a given preload. LVAD flow (CO_LVAD_) increases arterial pressure (AP) independent of LV ejection, thus shifts the E_a_ line upward by R · CO_LVAD_, where R is systemic resistance. This upward shift of the E_a_ line indicates the increases LV afterload, and, in turn, decreases SV (Figure 1A, solid loop). The reduction in SV (ΔSV) is geometrically derived as

(2) $$\begin{array}{l} \Delta SV\left(E_{es}{+E}_{a}\right)={R\;\cdot \;CO}_{LVAD} \end{array}$$

Rearranging Equation 2 gives

(3) $$\begin{array}{l} \Delta SV=\frac{R}{E_{es}+E_{a}}CO_{LVAD}=\frac{\frac{R}{T}}{E_{es}+E_{a}}T\;\cdot \;CO_{LVAD}=\\ \;\frac{E_{a}}{E_{es}+E_{a}}T\;\cdot \;CO_{LVAD}=\left(1-EF_{e}\right)T\;{\cdot \;CO}_{LVAD} \end{array}$$

where T is the cardiac cycle length. We define the effective ejection fraction (EF_e_) as the ratio of E_es_ to E_es_+E_a_. Dividing ΔSV by T yields the decrease in CO_LV_ (ΔCO_LV_) as

(4) $$\begin{array}{l} \Delta CO_{LV}=\left(1-EF_{e}\right)\;\cdot \;CO_{LVAD} \end{array}$$

Thus, the CO_NLV_ curve shifts downward under LVAD and CO curve through aortic valve under LVAD (CO_LV_) became following equation (Figure 1B);

(5) $$\begin{array}{r} CO_{LV}=CO_{NLV}-\left(1-EF_{e}\right)\;\cdot \;CO_{LVAD}\\ =S_{L}\left\{\ln\left(P_{LA}\right)+H_{L}\right\}-\left(1-EF_{e}\right)\;\cdot \;CO_{LVAD} \end{array}$$

By adding CO_LVAD_ to Equation 5, the total left ventricular CO (CO_TLV_) curve becomes as following (Figure 1B);

(6) $$\begin{array}{l} CO_{TLV}=S_{L}\left\{\ln\left(P_{LA}\right)+H_{L}\right\}-\left(1-EF_{e}\right)\;\cdot \;CO_{LVAD}+CO_{LVAD}\\ \;=S_{L}\left\{\ln\left(P_{LA}\right)+H_{L}\right\}+EF_{e}\;\cdot \;CO_{LVAD} \end{array}$$

### The Impact of LVAD on the RV Cardiac Output (CO_RV_) Surface

In the pulmonary circulation, LVAD does not directly impact the CO_RV_ surface. However, the effect of downstream pressure, P_LA_, is not negligible compared to pulmonary arterial pressure. Furthermore, LVAD significantly perturbs P_LA_. Therefore, we described the CO_RV_ as the surface, not the curve, as functions of P_LA_ and P_RA_.

(7) $$\begin{array}{l} CO_{RV}=S_{R}\left\{\ln\left(P_{RA}\right)+H_{R}\right\}-\alpha {\;\cdot \;P}_{LA} \end{array}$$

where S_R_, H_R_, and α are parameters of the right heart (see Supplementary Material in detail).

### The Impact of LVAD on the VR Surface

Since LVAD simply creates the LV-to-aorta bypass, LVAD does not change either the vascular properties or the stressed blood volume. Therefore, LVAD does not shift the VR surface or change its slopes. For the slopes of the VR surface, we used the values reported by Uemura et al. (15). Substituting those parameters into the equation of the VR surface yields

(8) $$\begin{array}{l} CO_{VR}=VR_{\max}-19.61P_{RA}-3.49P_{LA} \end{array}$$

where CO_VR_ is the amount of venous return, and VR_max_ is the maximum venous return.

**Figure 2:**
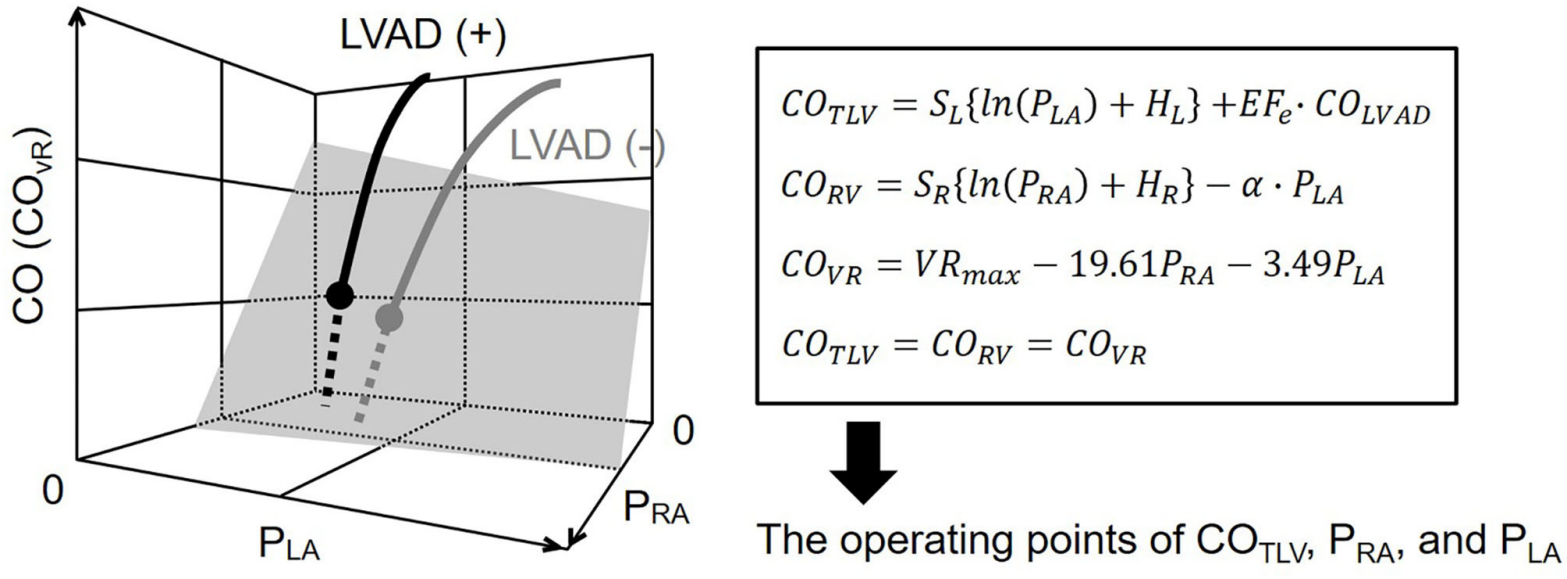
Diagram of circulatory equilibrium in the generalized Guyton's model. The intersection between the integrated CO curve (thick gray curve) and the venous return surface (shaded surface) represents cardiac output (CO), right atrial pressure (P_RA_), and left atrial pressure (P_LA_) at the circulatory equilibrium. The left ventricular assist device (LVAD) is incorporated into the framework of the generalized circulatory equilibrium. The LVAD shifts the native integrated CO curve upward. As a result, the intersection between the integrated CO curve and the VR surface moves upward (solid black circle). We numerically derived the equilibrium point on LVAD. S_L_, H_L_, are parameters of the left heart. S_R_, H_R_, and α are parameters of the right heart. VR_max_ is the maximum venous return. See detail in the Theoretical Consideration. CO, cardiac output; CO_TLV_, total cardiac output; CO_RV_, RV cardiac output; CO_VR_, amount of venous return; P_RA_, right atrial pressure; P_LA_, left atrial pressure; LVAD, left ventricular assist device, EF_e_, effective ejection fraction.

In the following experiments, we simultaneously solved Equations 6–8 and *CO*_*TLV*_=*CO*_*RV*_=*CO*_*VR*_, derived the operating points of CO, P_RA_, and P_LA_ (Figure 2), and compared them with those measured.

## Materials and Methods

### Preparation and Procedure

We used adult mongrel dogs of either gender weighing 16.9–22 kg (*n* = 11). Animal care was performed in strict accordance with the Guide for the Care and Use of Laboratory Animals by the US National Institutes of Health, and experiments were approved by the Committee on Ethics of Animal Experiment, Kyushu University Graduate School of Medical Sciences. All dogs were initially anesthetized with pentobarbital sodium (25 mg/kg) and vecuronium bromide (0.2 mg/kg). We then performed endotracheal intubation and started mechanical ventilation. We maintained an appropriate anesthesia level during the experiment by continuous infusion of isoflurane (1–2%) and pentobarbital sodium through a 5F catheter introduced into the right femoral vein during the experiment. We isolated the bilateral carotid sinuses and kept intra-sinus pressure constant at 100 mmHg to abolish the arterial baroreflex (16). We exposed the bilateral vagal trunks and cut them in the neck level to eliminate the vagally mediated buffering effects. Systemic arterial pressure (AP) was measured by a catheter-tipped micromanometer (model PC-751, Millar Instruments, Houston, TX) via the right common carotid artery. After a median sternotomy, fluid-filled catheters were placed in the left and right atria and connected to pressure transducers (model DX-360, Nihonkohden, Tokyo) to measure P_LA_ and P_RA_, respectively. We put an ultrasonic flowmeter (model PSB, Transonic, Ithaca, NY) around the ascending aorta to measure CO_LV_. We ligated the major branches and the first diagonal branch of the left anterior descending coronary artery (LAD), and added left circumflex coronary artery (LCx) ligation as needed to induce substantial worsening of LV function. After the condition was well-stabilized, we used a centrifugal pump (CBBPX-80, Medtronic, Minneapolis, MN) as LVAD (12). A systemic perfusion cannula was inserted in the left femoral artery. A draining cannula was placed in the left ventricle through the apex. We measured CO_LVAD_ by an in-line ultrasonic flow probe (model XN, Transonic, Ithaca, NY). We also inserted 5F catheter to left femoral vein to administer physiological saline as needed to keep mean AP above 70 mmHg for conducting 6–7 h experiment.

### Experimental Protocol (Figure 3)

#### Protocol 1: Hemodynamic Prediction by the Blood Volume Changing Determined CO Curve (n = 6)

Before LVAD support, we infused 250 ml of 10% dextran and waited to reach a steady-state of hemodynamics. We then withdrew blood stepwise at 2.5 ml/kg in each step (up to 4 or 5 steps) while recording P_LA_, P_RA_, and CO. We then estimated two-parameters (S_L_ and H_L_) in the CO_LV_-P_LA_ relation and three-parameters (S_R_, H_R_, and α) in the CO_RV_-P_RA_-P_LA_ relation by incorporating obtained hemodynamics into Equations 1 and 7 with the least-squares method.

**Figure 3:**
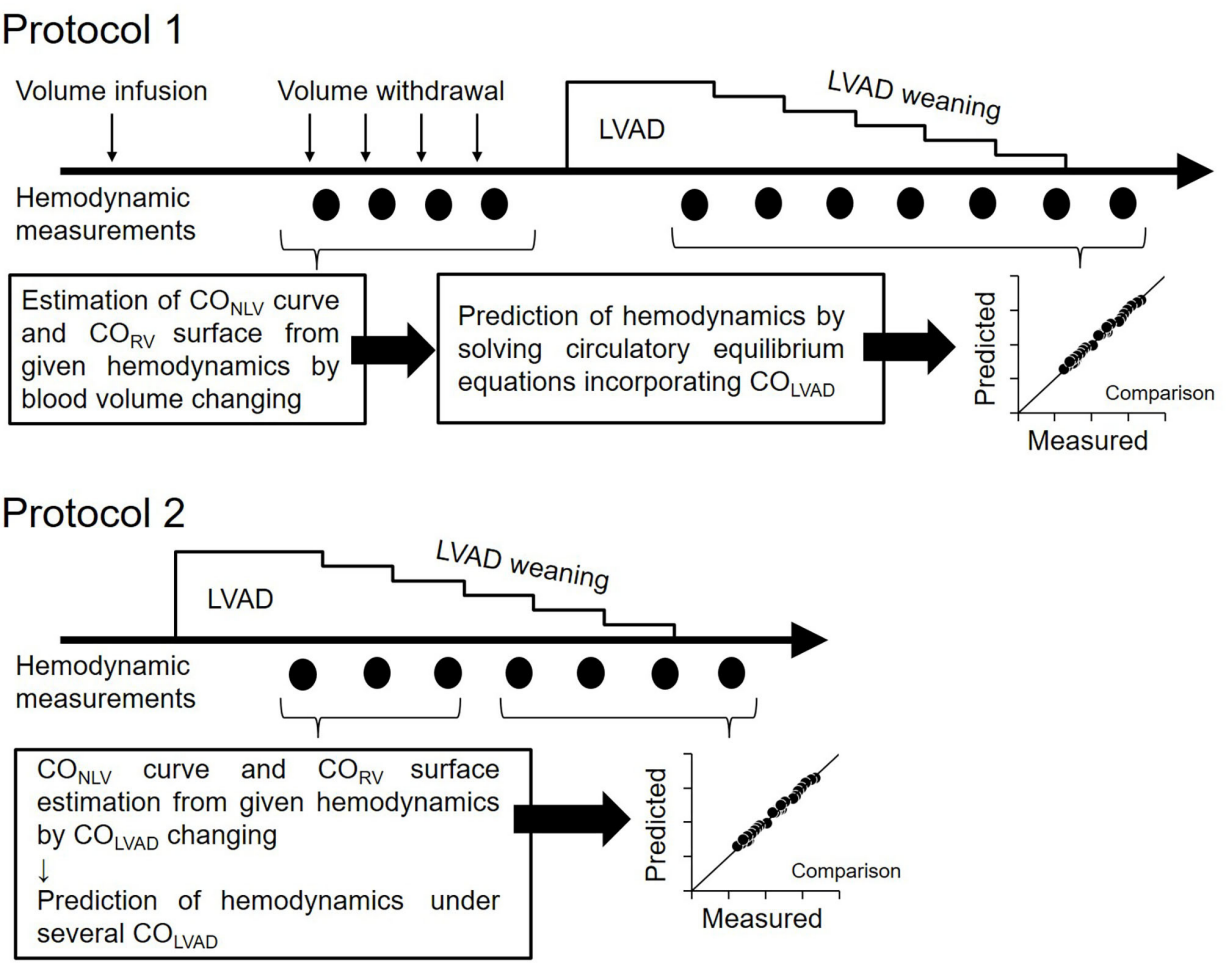
Protocol of this study. Protocol 1: Hemodynamic prediction by the blood volume changing determined CO curve. Protocol 2: Hemodynamic prediction by the CO_LVAD_ changing determined CO curve; LVAD, left ventricular assist device; CO_LVAD_, LVAD flow; CO_NLV_, native LV cardiac output through the aortic valve; CO_LV_, LV cardiac output under LVAD; CO_RV_, RV cardiac output.

After the CO curve estimation, we decreased CO_LVAD_ stepwise at 5 ml/min/kg in each step from ~70–110 ml/min/kg to 0 ml/min/kg and measured the P_LA_, P_RA_, and CO_LV_. Adding the CO_LVAD_ to the measured CO_LV_ yielded the total LV cardiac output, CO_TLV_, which equals CO_RV_, and venous return, CO_VR_. We then calculated the EF_e_ by substituting both the parameters of the CO_LV_ curve (S_L_ and H_L_) determined above and the equilibrated CO_LV_ and P_LA_ at the maximal CO_LVAD_ into Equation 4. We similarly obtained VR_max_ by substituting the CO_VR_, P_RA_, and P_LA_ at the maximal CO_LVAD_ into Equation 8. Assuming that EF_e_ and VR_max_ are constant irrespective of CO_LVAD_, we predicted CO_TLV_, P_RA_, and P_LA_ under various LVAD supports by simultaneously solving Equations 6–8. We compared the predicted hemodynamic values with those measured.

#### Protocol 2: Hemodynamic Prediction by the CO_LVAD_ Changing Determined CO Curve (n = 5)

The estimation of CO_LV_ curve and CO_RV_ surface by changing blood volume is impractical if not impossible in clinical settings. Thus, we employed the simplified estimation of hemodynamics on LVAD for another five dogs. We determined the parameters of the CO_LV_ curve and CO_RV_ surface under LVAD from three equilibrium points induced by the changes in CO_LVAD_. In other words, three sets of measured CO_LV_, P_LA_, and CO_LVAD_ uniquely determined S_L_, H_L_, and EF_e_ in Equation 5. We similarly estimated the S_R_, H_R_, and α by three sets of measured CO_RV_, P_RA_, and P_LA_ in Equation 7. After confirming that VR_max_ calculated in Equation 8 did not change despite the changes in CO_LVAD_, we predicted CO_TLV_, P_LA_, and P_RA_ under various LVAD supports from Equations 6–8 and *CO*_*TLV*_ =*CO*_*RV*_ =*CO*_*VR*_, and compared them with those measured. In this prediction, we excluded the data set that had been used to estimate the CO_LV_ curve and the CO_RV_ surface to avoid logical circularity.

### Data Analysis

All analog signals were digitized at 200 Hz using a 16-bit analog-to-digital converter (PowerLab 16/35, AD Instruments, Dunedin, New Zealand) with a dedicated laboratory computer system. Each data was averaged over 9 s and used for analysis after hemodynamic stability. Differences between groups were considered significant at *P* < 0.05 in paired *t*-test (Ekuseru-Toukei 2013; Social Survey Research Information Co. Ltd, Tokyo, Japan). We calculated the coefficient of determination (*r*^2^) for the goodness of fit and the standard error of estimate (SEE) for predictive accuracy.

## Results

### Baseline Hemodynamics (Protocol 1)

Table 1 showed the hemodynamics at baseline and after myocardial infarction (MI). The creation of MI significantly increased P_LA_ compared to baseline (*P* = 0.0019), indicating MI induced LV failure. In contrast, MI did not noticeably affect mean AP (MAP), heart rate (HR), CO, or P_RA_.

**Table 1:** The hemodynamics at baseline and after myocardial infarction (MI) in six dogs.

	**Baseline**					**After MI**				
	**MAP (mmHg)**	**HR (/min)**	**CO (ml/min/kg)**	**P_**LA**_ (mmHg)**	**P_**RA**_ (mmHg)**	**MAP (mmHg)**	**HR (/min)**	**CO (ml/min/kg)**	**P_**LA**_ (mmHg)**	**P_**RA**_ (mmHg)**
1	139	156	76	9.1	6.5	115	133	77	14.4	6.9
2	92	167	113	8.1	4.2	82	132	121	16.3	6.4
3	161	161	105	8.8	3.6	73	176	62	18.1	3.6
4	110	123	103	4.8	2.6	98	86	119	9.4	4.1
5	93	178	117	9.3	6.8	96	155	144	18.6	8.1
6	107	152	103	6.5	3.2	105	141	86.6	9.3	3.7
	117 (28)	156 (19)	103 (14)	7.75 (1.8)	4.48 (1.8)	95 (15)	137 (30)	102 (31)	14.4^*^ (4.1)	5.5 (1.9)

*Bottom column represents the average and standard deviation value of six dogs in protocol 1*.

* *P < 0.05, vs. Baseline. MI, myocardial infarction; MAP, mean arterial pressure; HR, heart rate; CO, cardiac output; P_LA_, left atrial pressure; P_RA_, right atrial pressure*.

### Determination of CO_LV_ Curve and CO_RV_ Surface by Changing Blood Volume (Protocol 1)

**Figure 4:**
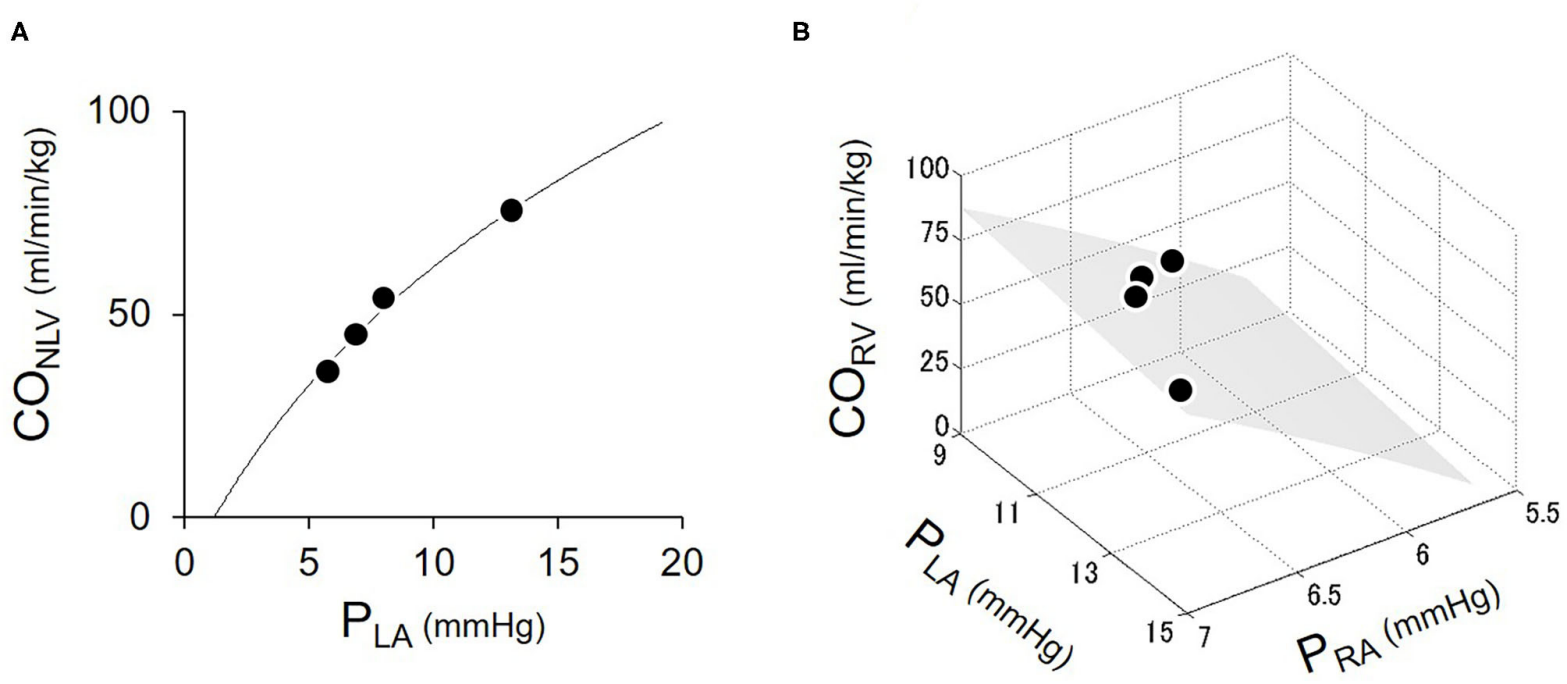
Estimation of LV cardiac output (CO_NLV_) curve **(A)** and RV cardiac output (CO_RV_) surface **(B)**. Open circles were the measured values obtaining by changing blood volume. The solid curve represented the fitted logarithmic curve. The shaded surface represents the fitted CO_RV_ surface. CO_NLV_, native LV cardiac output through the aortic valve; CO_RV_, RV cardiac output; P_RA_, right atrial pressure; P_LA_, left atrial pressure.

Figure 4 shows the representative cardiac output data where we fitted logarithmic functions to the measured values obtaining by changing blood volume. As shown in Figure 4A, increases in P_LA_ increased CO_NLV_. A two-parameters logarithmic function approximates the CO_NLV_ curve (Equation 1) reasonably well. Figure 4B illustrates CO_RV_ in response to changes in P_RA_ and P_LA_. Increases in P_RA_ increased CO_RV_, while increases in P_LA_ decreased CO_RV_. A surface generated by the three-parameters logarithmic function of P_RA_ and P_LA_ (CO_RV_ surface, Equation 7) approximated the changes in CO_RV_ reasonably well.

Table 2 summarized the parameters of CO_NLV_ curves and CO_RV_ surfaces in all six dogs. The fact that the coefficient of determination was quite high (*r*^2^ = 0.94–0.99) in each dog suggested that the 2-parameter function and 3-parameter function accurately represent the CO_NLV_ curve and CO_RV_ surface, respectively.

**Table 2:** The estimated parameters of native left-heart cardiac output (CO_NLV_) curve and right-heart cardiac output (CO_RV_) surface by changing blood volume.

	**CO**_****NLV****_ **curve**			**CO**_****RV****_ **surface**			
	**S_**L**_** **(ml/min/kg)**	**H_**L**_** **(unitless)**	***r^**2**^***	**S_**R**_** **(ml/min/kg)**	**H_**R**_** **(unitless)**	**α** **(ml/min/kg/mmHg)**	***r^**2**^***
1	80.8	−1.7	0.99	332	−1.64	1.77	0.98
2	41.3	0.15	0.95	171	−0.916	2.46	0.94
3	26.8	−0.5	0.94	137	−0.65	1.35	0.97
4	71.4	−0.61	0.98	138	−0.32	0.69	0.99
5	85.5	−1.2	0.94	664	−1.68	6.93	0.99
6	48.1	−1.21	0.95	261	−1.09	4.14	0.97
	59 (23.7)	−0.845 (0.656)		284 (201)	−1.05 (0.54)	2.89 (2.3)	

*Bottom column represents the average and standard deviation value of six dogs in protocol 1*.

*S_L_ and H_L_ are the parameters of native left heart. S_R_, H_R_, and α are those of right heart (see Supplementary Materials). r^2^ is coefficient of determination*.

*CO_NLV_, the native LV cardiac output through the aortic valve; CO_RV_, RV cardiac output*.

### Prediction of Hemodynamics Under Various Levels of LVAD Support (Protocol 1)

**Figure 5:**
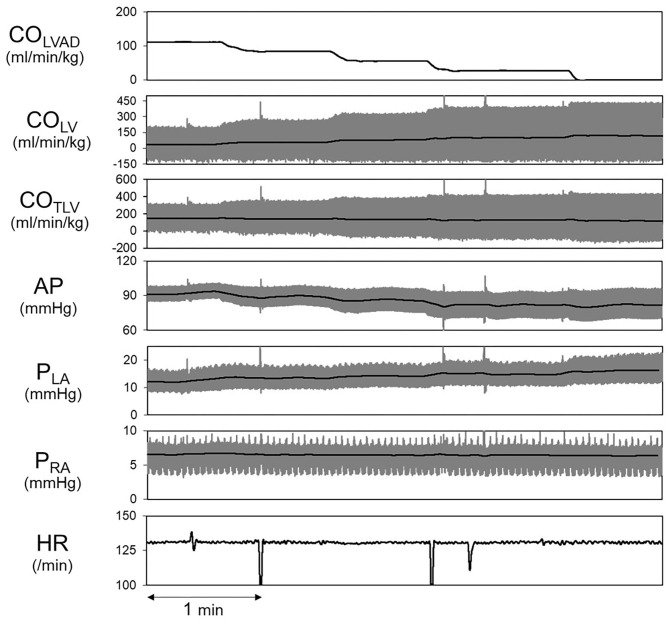
Hemodynamic changes induced by decreasing CO_LVAD_ in a representative animal. The thick black lines indicate the averages, and the thin gray lines indicate instantaneous data. LVAD, left ventricular assist device; CO_LVAD_, LVAD flow; CO_LV_, LV cardiac output under LVAD; CO_TLV_, total cardiac output; AP, arterial pressure; P_LA_, left atrial pressure; P_RA_, right atrial pressure; HR, heart rate.

**Figure 6:**
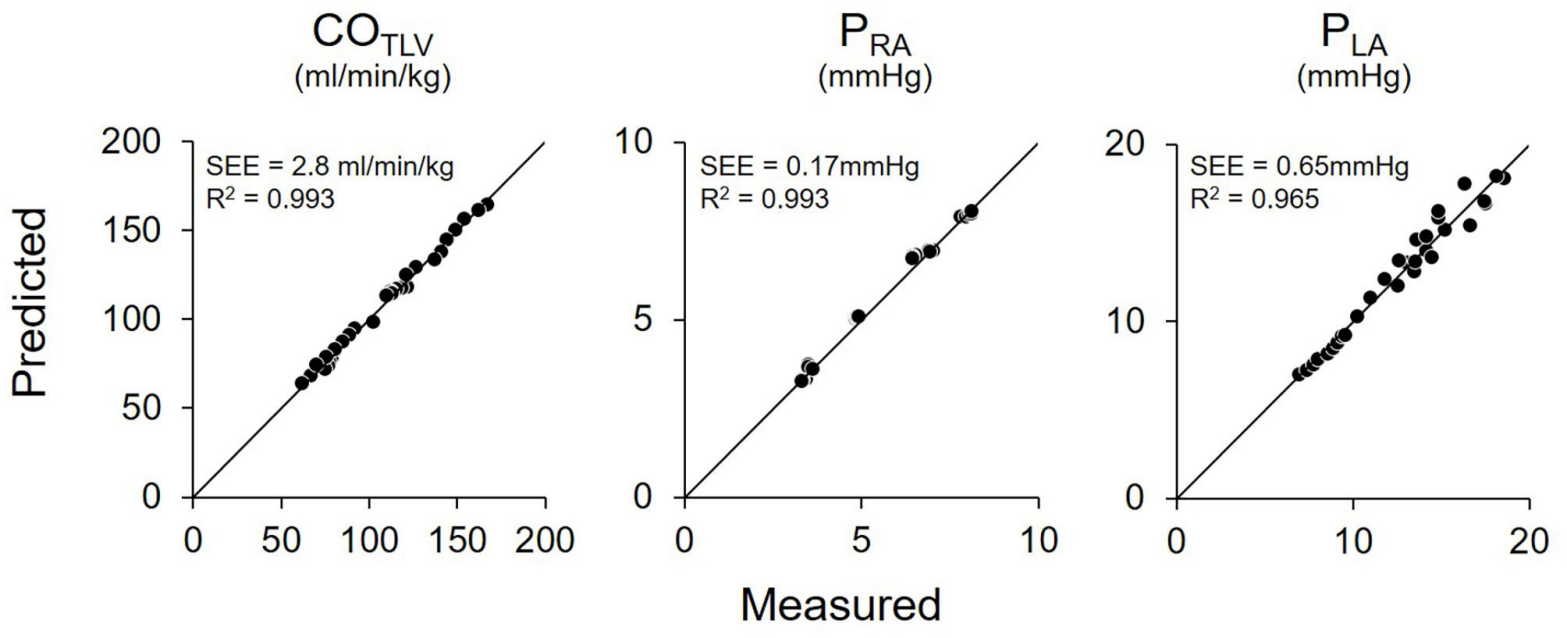
Relationship between predicted and measured in total cardiac output (CO_TLV_), right atrial pressure (P_RA_), and left atrial pressure (P_LA_) in pooled data in Protocol 1. The predicted values matched well with those measured. The thin black lines denote the lines of identity. SEE, standard error of estimate; r^2^, coefficient of determination.

Shown in Figure 5 is the time series of hemodynamics in response to the stepwise decrease in CO_LVAD_. Decreases in CO_LVAD_ increased CO_LV_ and P_LA_, while decreased the CO_TLV_ and AP. Despite changes in AP, HR remained unchanged because of the abolishment of the baroreflex. Table 3 shows the estimated EF_e_ and VR_max_ for each dog. As we expected, the creation of MI markedly lowered EF_e_. VR_max_ varied among dogs, indicating the variability of stressed blood volume. Figure 6 demonstrates the relationship between predicted and measured CO_TLV_, P_RA_, and P_LA_ in all dogs. Regression analysis revealed that the predicted CO_TLV_ (y = 0.983x + 2.845, *r*^2^ = 0.993, SEE = 2.8 ml/min/kg), P_RA_ (y = 1.00x + 0.0824, *r*^2^ = 0.993, SEE = 0.17 mmHg), and P_LA_ (y = 1.01x−0.0728, *r*^2^ = 0.965, SEE = 0.65 mmHg) matched well with those measured.

**Table 3:** The calculated effective ejection fraction (EF_e_) and maximum venous return (VR_max_).

	**EF_**e**_** **(unitless)**	**VR_**max**_** **(ml/min/kg)**
1	0.30	257
2	0.30	319
3	0.27	199
4	0.25	210
5	0.48	366
6	0.55	230
	0.37 (0.13)	264 (66)

*Bottom column represents the average and standard deviation value of six dogs in protocol 1*.

*EF_e_, effective ejection fraction; VR_max_, maximum venous return*.

### Simplified Prediction of Hemodynamics Under Various Levels of LVAD Support (Protocol 2)

**Figure 7:**
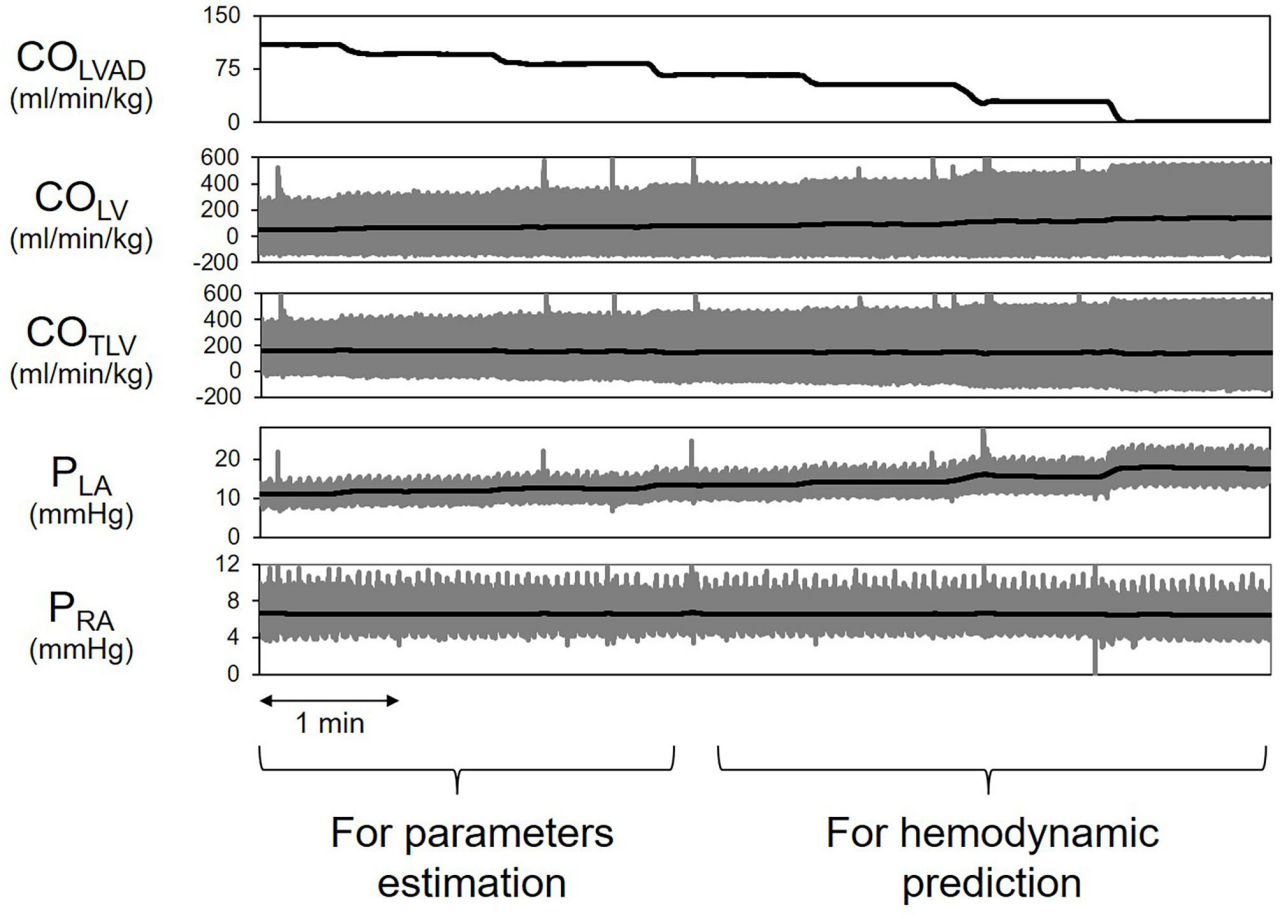
Simplified prediction of circulatory equilibrium in an animal. The measurement of three equilibrium points for given left ventricular assist device flow (CO_LVAD_) enabled us to estimate the native LV cardiac output (CO_NLV_) curve, the RV cardiac output (CO_RV_) surface, and the VR surface. We then numerically predicted the circulatory equilibrium points for the various CO_LVAD_ values. LVAD, left ventricular assist device; CO_LVAD_, LVAD flow; CO_LV_, LV cardiac output through the aortic valve under LVAD; CO_TLV_, total CO; AP, arterial pressure; P_LA_, left atrial pressure; P_RA_, right atrial pressure.

**Figure 8:**
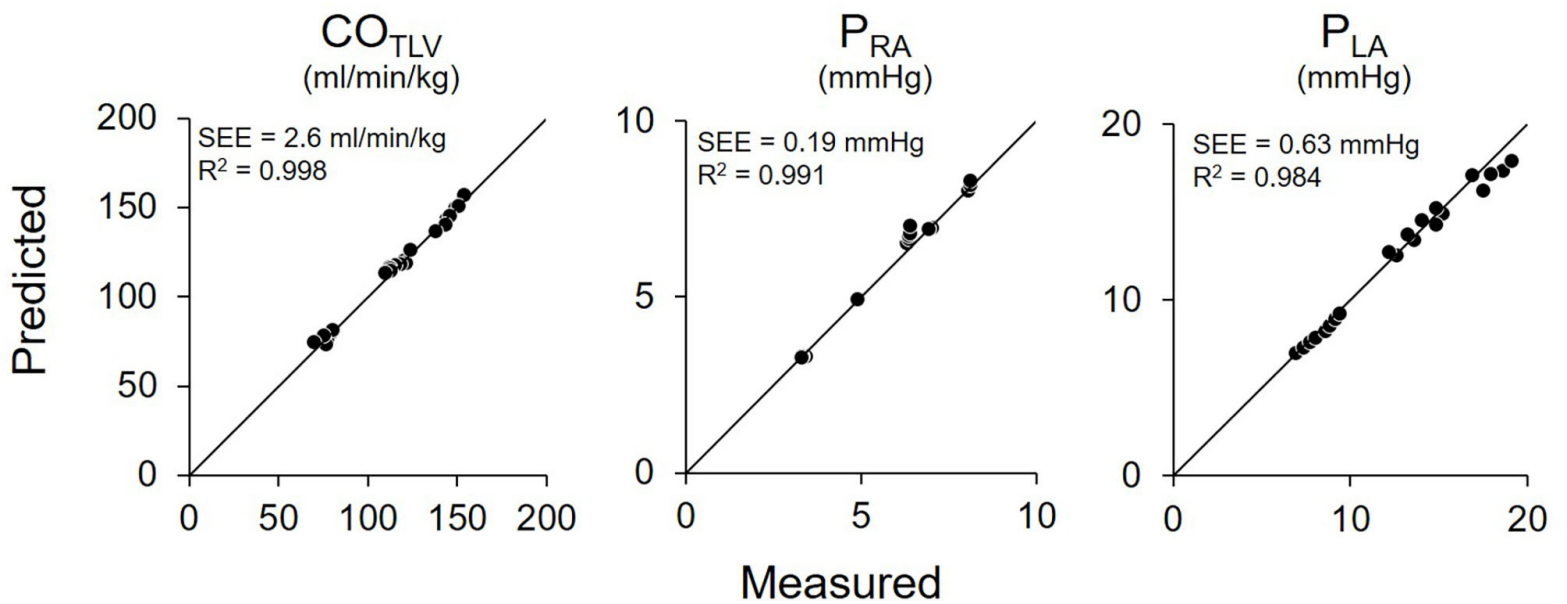
Pooled data of predicted and measured total cardiac output (CO_TLV_), right atrial pressure (P_RA_), and left atrial pressure (P_LA_) in Protocol 2. Predicted CO_TLV_, P_RA_, and P_LA_ values correlated well with those measured. The thin black lines are the lines of identity. SEE, standard error of estimate; r^2^, coefficient of determination.

Figure 7 shows the representative time series of hemodynamics under several CO_LVAD_ levels. By using three points hemodynamic data in each dog (Table 4), we estimated the parameters of the CO_NLV_ curve, the CO_RV_ surface, and the VR surface under LVAD support as shown in Table 5. We then predicted CO_TLV_, P_RA_, and P_LA_ from the data set that had not been used to estimate the CO_NLV_ curve, the CO_RV_ surface, or the VR surface. The predicted CO_TLV_, P_RA_, and P_LA_ correlated well with those measured (Figure 8). Regression analysis of CO_TLV_ (y = 0.984x + 3.40, *r*^2^ = 0.998, SEE = 2.57 ml/min/kg), P_RA_ (y = 1.04x-0.125, *r*^2^ = 0.991, SEE = 0.20 mmHg) and P_LA_ (y = 0.925x + 0.721, *r*^2^ = 0.984, SEE = 0.634 mmHg) demonstrated the good agreement between the predicted and measured.

**Table 4:** The hemodynamics response to LVAD flow changes for parameters estimation in protocol 2.

		**Baseline**	**Step 1**	**Step 2**
1	CO_LVAD_ (ml/min/kg)	122	117	112
	CO_LV_ (ml/min/kg)	10	12	19
	P_LA_ (mmHg)	6.7	6.7	6.7
	P_RA_ (mmHg)	3.4	3.4	3.4
2	CO_LVAD_ (ml/min/kg)	104	90	75
	CO_LV_ (ml/min/kg)	147	159	170
	P_LA_ (mmHg)	13.3	14.0	14.7
	P_RA_ (mmHg)	7.1	7.2	7.2
3	CO_LVAD_ (ml/min/kg)	108	94	80
	CO_LV_ (ml/min/kg)	56	66	74
	P_LA_ (mmHg)	10.5	11.2	11.9
	P_RA_ (mmHg)	6.3	6.4	6.4
4	CO_LVAD_ (ml/min/kg)	75	59	44
	CO_LV_ (ml/min/kg)	17	29	40
	P_LA_ (mmHg)	10.2	11.0	11.8
	P_RA_ (mmHg)	4.8	4.9	4.9
5	CO_LVAD_ (ml/min/kg)	110	96	81
	CO_LV_ (ml/min/kg)	59	71	81
	P_LA_ (mmHg)	12.9	13.0	14.1
	P_RA_ (mmHg)	7.7	7.8	7.9

*Left column indicates the individual identification number of dog in protocol 2. We changed LVAD flow to estimate the parameters of native CO_NLV_ curve, CO_R_ surface and venous return surface. By using the estimated parameters, S_L_, H_L_, S_R_, H_R_, a, EF_e_, VR_max_, we then predicted hemodynamics during LVAD weaning*.

*CO_LVAD_, LVAD flow; CO_LV_, LV cardiac output under LVAD; P_LA_, left atrial pressure; P_RA_, right atrial pressure*.

**Table 5:** The estimated parameters of native left-heart cardiac output (CO_NLV_) curve, right-heart cardiac output (CO_RV_) surface and venous return (VR) surface by changing LVAD flow in protocol 2.

	**CO**_****NLV****_ **curve**				**CO**_****RV****_ **surface**				**VR_**max**_** **(ml/min/kg)**
	**S_**L**_** **(ml/min/kg)**	**H_**L**_** **(unitless)**	**EF_**e**_** **(unitless)**	***r^**2**^***	**S_**R**_** **(ml/min/kg)**	**H_**R**_** **(unitless)**	**α** **(ml/min/kg/mmHg)**	***r^**2**^***	
1	38.5	0.43	0.43	0.97	85.2	0.84	7.44	0.99	212
2	99.6	−0.64	0.54	0.99	146	0.18	4.75	0.98	473
3	50.0	−0.31	0.58	0.99	149	−0.14	8.72	0.99	323
4	89.4	−1.83	0.64	0.99	96.2	−0.12	4.67	0.99	222
5	23.0	3.30	0.32	0.99	50.0	2.90	6.15	0.99	366
	60.1 (33.0)	0.19 (1.92)	0.50 (0.13)		105.3 (42.2)	0.73 (1.28)	6.35 (1.75)		319.2 (108.2)

*Bottom column represents the average and standard deviation value of five dogs in protocol 2*.

*S_L_ and H_L_ are the parameters of native left heart. S_R_, H_R_, and α are those of right heart (see Supplementary Materials). r^2^ is coefficient of determination. EF_e_ and VR_max_ are effective ejection fraction and maximum venous return (VR), respectively*.

*CO_NLV_, the native LV cardiac output through the aortic valve; CO_RV_, RV cardiac output*.

## Discussion

In this study, we analyzed the LVAD interaction with the native LV cardiac output in determining total LV cardiac output. We then developed a framework to predict the impact of LVAD on hemodynamics. In Protocol 1, we showed that we could predict hemodynamics on LVAD by determining the CO_NLV_ curve and the CO_RV_ surface with a volume-changing technique. Furthermore, in Protocol 2, the hemodynamic assessment during the small perturbations of CO_LVAD_ enabled us to predict the CO_TLV_, P_LA_, and P_RA_ when LVAD was weaned.

The most critical result of this study is that the framework of generalized circulatory equilibrium can quantitatively predict the hemodynamic impact of LVAD. We previously reported the impact of total LVAD support on the framework (12). Considering various situations under LVAD, especially LVAD weaning after cardiac recovery, we need to expand this framework to the partial LVAD support. As can be seen in Equation 6 and Figure 1B, the LVAD shifts the CO_TLV_ curve upward by EF_e_ · CO_LVAD_, indicating the poorer LV function, the poorer increases in CO_TLV_. The depressed LV is more susceptible to the LVAD-induced increases in LV afterload; that is, LVAD decreases the SV more in low LV contractility than in high LV contractility (Equation 3). In addition, the results of a computational study by using a multi-element cardiovascular model were in line with our results (17). Our framework, in which we incorporated LVAD in the generalized circulatory equilibrium and ventricular-arterial coupling, can algebraically define the cardiovascular system and its equilibrium. This makes the impact of LVAD on CO_TLV_ physiologically interpretable such as the reduction of CO_NLV_ equals (1-EF_e_) · CO_LVAD_. The multi-element cardiovascular model cannot easily attribute a particular observation to a specific element of the system. Despite small increases in CO_TLV_ in our study, we found the significant decreases in P_LA_ from 19 to 7 mmHg (Figure 6). In the framework of circulatory equilibrium, LV failure flattens the slope of the CO_TLV_ curve (18). Thereby, given that LVAD does not change the VR surface, the small upward-shift of the flattened CO_TLV_ curve results in more substantial decreases in P_LA_ compared with the steeper CO_TLV_ curve. Thus, the framework of generalized circulatory equilibrium is robust in understanding and predicting hemodynamics on LVAD.

In our framework, we need to use EF_e_ to incorporate the LVAD effects into circulatory equilibrium. We defined EF_e_ as the ratio of E_es_ to (E_es_+E_a_) (Equation 3). This is equivalent to say that EF_e_ equals SV divided by end-diastolic volume in excess of V_0_, where V_0_ is the volume axis intercept of the end-systolic pressure-volume relationship (19). Since E_es_ characterizes the ventricular chamber property, and E_a_ characterizes the arterial property, CO_LVAD_ cannot change these properties. In this sense, we need to carefully interpret standard ejection fraction (EF), calculated by echocardiogram, under LVAD support. Because EF is the ratio of SV to end-diastolic volume (V_ed_), EF could markedly change with LVAD support. Furthermore, regional ischemia significantly increases the V_0_ (20), which makes the difference between EF and EF_e_ even more extensive in our acute MI preparation. For these reasons, we used EF_e_, not EF, for prediction in the present study.

We previously reported the impact of extracorporeal membrane oxygenation (ECMO) on circulatory equilibrium and showed that ECMO also suppresses the CO_NLV_ curve by (1-EF_e_) · F_ECMO_, where F_ECMO_ indicates the ECMO flow (21). In terms of the shift in E_a_ line, LAVD and ECMO are the same impacts in increasing afterload as long as the support flow is the same. Since LVAD can shift the CO_TLV_ curve upward by adding the LVAD flow on a decreased CO_LV_ curve, LVAD decreases P_LA_. ECMO increases total systemic flow, while ECMO increases LV afterload, decreases the native CO curve (=total CO curve), and results in increases of P_LA_.

### Clinical Implication

As we discussed above, the LVAD shifts the CO_TLV_ curve upward by EF_e_ · CO_LVAD_, indicating the poorer LV function, the poorer increases in CO_TLV_. This relationship may become important for the management of transvascular LVAD. Impella 2.5 or sometimes CP cannot necessarily generate sufficient flow to establish total support where LV is no longer ejecting (22). These transvascular LVADs have often been used for cardiogenic shock (23). Since the lower EF_e_ reduces the LVAD increase of CO_TLV_, the hemodynamic benefit of transvascular LVADs is limited in patients with severe LV dysfunction.

The present study suggested that, with small changes in CO_LVAD_, we could identify the native LV cardiac output curve (CO_NLV_ curve), EF_e_ in LV, CO_RV_ surface, and VR surface as shown in Table 5. The values of parameters were acceptable, while these were different between protocols 1 and 2 because of the differences in dogs, the way of volume changing and parameters estimation. Thus, we need to interpret each parameter carefully. We have validated the accuracy of hemodynamic prediction (CO, P_RA_, and P_LA_), while we did not compare the parameters calculated by our proposed equation to those of direct measurements. Further investigations might be needed to evaluate the utility of this method in terms of the estimation of cardio-vascular properties. The proposed framework allows us to predict the hemodynamics even after LVAD weaning. Since the hemodynamic assessment during “off-pump” is critical for patients undergoing LVAD removal (11), the proposed framework would be useful to predict the hemodynamic changes after LVAD removal without switching off LVAD. The present framework does not only elude the risk of thrombosis associated with “off-pump” (24) but also distinguish the patients who might deteriorate to heart failure after LVAD weaning in advance. Further clinical investigations might be needed to evaluate the utility of this hemodynamic prediction method.

### Limitations

There are several limitations in our study. First, we conducted experiments using anesthetized and open-chest dogs. Furthermore, we isolated the bilateral carotid sinuses and cut the vagal trunks. Since both the baroreflex and other reflexes through the vagal nerves alter the vascular as well as cardiac properties (16), we eliminated those reflexes to clarify the isolated impacts of LVAD on hemodynamics. Second, there was some variability of the maximal CO_LVAD_ among dogs in our study. It may well be attributed to the fact that we conducted the experiment where LV remained ejecting under LVAD support. It means that the degree of MI affected how much we could increase CO_LVAD_. LV would quickly become the non-ejecting state as we increase CO_LVAD_ if MI severely impairs LV contractility, indicating that the variability of maximal CO_LVAD_ under the LV ejecting condition depends on MI size. Third, we did not measure either the pressure-volume loop or echocardiogram. Although the EF_e_ we used in the equation is different from standard EF calculated by echocardiogram as we addressed in the discussion, the standard EF as well as the direct measured EF_e_ by pressure-volume loop in the same dog may help the interpretation of EF_e_ obtained from the equation. Further detailed experiment might be needed to clarify the accuracy of our method in estimating EF_e_. Fourth, our proposed framework is a static mathematical model of circulation. Thus, we did not consider dynamic hemodynamic change during the cardiac cycle in LV under LVAD support. To improve the estimation accuracy of hemodynamics, we need to adopt the fluid dynamics and dynamic change in cardio-vascular properties into our framework. Lastly, we utilized the previously reported values as the slopes of the VR surface for simplicity (15). Needless to say, the slopes of the VR surface in humans have yet to be investigated. Therefore, all of them might affect the results, especially when we predict the effect of LVAD on hemodynamics in awake and closed-chest humans with intact reflexes.

## Conclusions

The proposed framework is capable of quantitatively predicting the hemodynamic impact of partial LVAD support. Circulatory equilibrium is generalizable and essential for understanding the cardiovascular system, including LVAD. It would provide the physiological insight into hemodynamics on LVAD and contribute to the safe management of patients with LVAD.

## Data Availability Statement

The datasets generated for this study are available on request to the corresponding author.

## Ethics Statement

The animal study was reviewed and approved by the Committee on Ethics of Animal Experiment, Kyushu University Graduate School of Medical Sciences.

## Author Contributions

TK, KSa, TN, and KSu conceived of the presented idea and designed the study. TK and KSa performed the data collection. TK, KSa, and TN performed the analysis and took the lead in writing the manuscript. TK, KSa, and KSu edited and revised manuscript. All authors discussed the results and contributed to the final manuscript. All authors approved the final version of the manuscript and agree to be accountable for the study.

## Conflict of Interest

KSa received research funding from Omron Healthcare Co., Abiomed Japan K.K., and Zeon Medical Inc., and honoraria from Abiomed Japan K.K. KSu received research funding from Omron Healthcare Co. and honoraria from Abiomed Japan K.K. The remaining authors declare that the research was conducted in the absence of any commercial or financial relationships that could be construed as a potential conflict of interest. The handling editor declared a past co-authorship with one of the authors KSu.
