# Supplementary material for: The Partial Support of the Left Ventricular Assist Device Shifts the Systemic Cardiac Output Curve Upward in Proportion to the Effective Left Ventricular Ejection Fraction in Pressure-Volume Loop
Source: Front Cardiovasc Med. 2020 Sep 15;7:163. doi: 10.3389/fcvm.2020.00163 (PMC7522370; doi:10.3389/fcvm.2020.00163)
Supplement: Supplementary file 1 [file Data_Sheet_1.DOCX]

Supplementary Material

***Derivation of CO curve and surface***

Uemura et al previously derived the mathematical model of the CO curve (Uemura et al., 2004). Briefly, in the framework of ventricular arterial coupling, the ventricular property is characterized by E_es_.

$$\begin{aligned} P_{es}=E_{es}\left( V_{es}-V_{0} \right){=E}_{es}\left( V_{ed}-SV-V_{0} \right) \#\left( S1 \right) \end{aligned}$$

where P_es_ and V_es_ is end-systolic pressure and volume of ventricle, respectively. In contrast, the relationship of MAP and mean flow in the arterial system is expressed as follows.

$$\begin{aligned} MAP=R\cdot mean flow \#\left( S2 \right) \end{aligned}$$

where R is the systemic vascular resistance. Rearranging Eq. S2 yields E_a_ which is expressed by R divided by cardiac cycle length, T.

$$\begin{aligned} {MAP=\frac{R}{T}\cdot SV=E}_{a}\cdot SV\#\left( 3S \right) \end{aligned}$$

When we assume that MAP approximates P_es_, SV is given as a function of V_ed_ from Eqs. S1 and S3 as follows (Sunagawa et al., 1984).

$\begin{aligned} MAP \cong P_{es} \leftrightarrow SV=\frac{E_{es}}{E_{es}+E_{a}}\left( V_{ed}-V_{0} \right)\#\left( S4 \right) \end{aligned}$

On the other hand, end-diastolic pressure volume relationship is known to be exponential.

$$\begin{aligned} P_{ed}=\alpha e^{{kV}_{ed}}\#\left( S5 \right) \end{aligned}$$

where P_ed_ is ventricular end-diastolic pressure and k and α are constant parameters of ventricle.

Substituting Eq. S5 into Eq. S4 and rearranging yields CO as logarithmic function as shown in Eq. S6.

$$\begin{aligned} CO=\frac{1}{k}\cdot\frac{1}{T}\cdot\frac{E_{es}}{E_{es}+E_{a}}\left\{ \ln\left( P_{ed} \right)+\ln\left( \frac{1}{\alpha} \right)-kV_{0} \right\}\#\left( S6 \right) \end{aligned}$$

P_ed_ can be expressed by mean atrial pressure as follows.

$$\begin{aligned} P_{ed}=\beta P_{AT} \#\left( S7 \right) \end{aligned}$$

where P_AT_ represents mean atrial pressure and β is constant parameter of the relationship between P_ed_ and P_AT_. Substituting Eq. S7 into Eq. S6 gives Eq. S8.

$$\begin{aligned} CO=\frac{1}{k}\cdot\frac{1}{T}\cdot\frac{E_{es}}{E_{es}+E_{a}}\left\{ \ln\left( P_{AT} \right)+\ln\left( \frac{\beta}{\alpha} \right)-kV_{0} \right\}\#\left( S8 \right) \end{aligned}$$

Therefore, we can simplify Eq. S8 with two parameters, S and H, as shown in Eq. S9.

$$\begin{aligned} CO=S\left\{ \ln\left( P_{AT} \right)+H \right\}\#\left( S9 \right) \end{aligned}$$

Although Uemura et al. used the three-logarithmic functions for the CO curve in their paper, we chose two-logarithmic functions for simplicity (Sakamoto et al., 2015).

***The incorporation of downstream pressure into right CO surface***

In right CO surface, the impact of downstream pressure (=P_LA_) is not negligible. Incorporating downstream pressure (=P_LA_) into Eq. S9, we can algebraically derive the following equation (Sakamoto et al., 2015).

$$\begin{aligned} CO_{RV}=S_{R}\left\{ \ln\left( P_{RA} \right)+H_{R} \right\}-\frac{P_{LA}}{{TE}_{es}+R_{p}} \#\left( S10 \right) \end{aligned}$$

where E_es_ is the right ventricular systolic property and R_p_ is the pulmonary vascular resistance, respectively. Thus, right CO surface can be represented as following;

$$\begin{aligned} {CO}_{RV}=S_{R}\left\{ \ln\left( P_{RA} \right)+H_{R} \right\}-\alpha P_{LA}\#\left( S11 \right) \end{aligned}$$

where α is the coefficient of downstream pressure for right heart.

***References***

Uemura K, Sugimachi M, Kawada T, Kamiya A, Jin Y, Kashihara K, Sunagawa K. (2004) A novel framework of circulatory equilibrium. *Am J Physiol.* 286: H2376-H2385.

Sunagawa K, Sagawa K, Maughan WL. (1984) Ventricular interaction with the loading system. *Ann Biomed Eng.* 12: 163-189.

Sakamoto K, Saku K, Kishi T, Kakino T, Tanaka A, Sakamoto T, Ide T, Sunagawa K. (2015) Prediction of the impact of venoarterial extracorporeal membrane oxygenation on hemodynamics. *Am J Physiol Heart Circ Physiol.* 308: H921-30.
